# Adaptive Behavior in Slovak Children with Intellectual Disability in Institutional Care

**DOI:** 10.3390/children9121911

**Published:** 2022-12-06

**Authors:** Margaréta Hapčová, Hana Celušáková, Daniela Turoňová, Michaela Souček Vaňová, Lenka Besedová, Diana Demkaninová, Katarína Babinská

**Affiliations:** 1 Department of Psychology, Faculty of Arts, Comenius University, 811 02 Bratislava, Slovakia; 2 Institute of Physiology, Faculty of Medicine, Comenius University, 813 72 Bratislava, Slovakia; 3 Department of Psychology, Faculty of Education, University of Matej Bel, 974 11 Banská Bystrica, Slovakia; 4 Center for Educational and Psychological Counseling and Prevention, 811 04 Bratislava, Slovakia

**Keywords:** adaptive behavior, intellectual disability, institutional care, peripartum risk factors, postnatal risk factors, VABS-3

## Abstract

This study aimed to analyze the adaptive skills of children with intellectual disabilities in institutional care. We focused on communication, socialization, daily living skills and their relationship with risk factors, and institutional care. Our sample included 197 children aged 5–18 years (M = 12.8, SD = 2.97), 50% boys, with IQ < 85 placed in different types and lengths of stay in institutional care. There were 17% that presented with borderline intellectual functioning (IQ 84–87) and 83% that had intellect disabilities. Adaptive behavior (AB) was assessed by Vineland Adaptive Behavior Scale (VABS-3). The BIF and Mild ID groups did not differ in Socialization. The profile of adaptive behavior for BIF and Mild ID was Daily Living Skills > Communication > Socialization, and for Moderate and Severe ID, Socialization > Daily Living Skills > Communication. Longer institutional care was associated with lower competencies in AB. Gender differences were found, females overperformed males in Socialization, Daily Living Skills, and ABC score. Levels of ID, gender, length of stay in institutional care, and neonatal difficulties were significant predictors in the model which explain the 63% variance of AB. The practical implications of the results are discussed related to the assessment of ID, prevention, and care for institutionalized children.

## 1. Introduction

When reflecting on the adaptive skills of children with intellectual disabilities (ID) who are in institutional care, it is necessary to consider various factors. They include intellectual disabilities that are associated with the decline in adaptive behavior, their level depending on the ID severity [1,2,3]. The presence of co-occurring conditions is associated with changes in adaptive behavior [4,5,6]; furthermore, maltreatment before being placed in institutional care could have a negative impact on current adaptive behavior [7,8,9]. Finally, current institutional care is associated with specific conditions that may have a negative impact on adaptive behavior [4,10,11].

Adaptive behavior (AB) refers to abilities and skills that people learn throughout life and that allow them to function independently in society. AB is age-appropriate behavior, defined according to the expectations or standards of society [12]. The level of adaptive behavior reflects how much support a person needs in order to lead an independent life. The complexity of adaptive behavior increases with age [13,14,15], but the development of adaptive skills may not be uniform in all children. Gender differences have been described in several studies, but they explain only a small amount of variance. Moreover, it seems that they occur at different ages in different domains of adaptive behavior. On the other hand, girls with neurodevelopmental conditions (e.g., autism spectrum disorder—ASD, attention deficit hyperactivity disorder—ADHD) appear to have higher adaptive scores than their male peers [16,17]. The educational and social environment provides a stimulus and an opportunity to acquire skills [18].

It has been shown that AB is tightly related to intellectual disability (ID). It is assumed that the level of AB declines with decreasing intellectual functioning [3]. In accordance with DSM-5 and ICD-11 criteria, intellectual disability is defined by a decrease in intellectual and adaptive functioning two or more standard deviations of the score below the mean [2,3]. The ID severity level is classified based on the degree of support needed [2]. According to the National Academies of Sciences, Engineering, and Medicine [19], individuals with a mild level of ID represent the largest subgroup of ID (about 85%). Populations with mild disabilities are generally slower in conceptual development, social skills, and daily functioning skills. They can learn practical skills that will allow them to live independently or with minimal support. This group can achieve literacy and partial economic and social independence in adulthood [20], but they are at risk of burnout, especially with full-time work and low job satisfaction [21]. The development of individuals with a moderate level of ID (making up 10% of ID) varies but it is generally limited to basic skills. Considerable and consistent support is needed for their independent functioning in adulthood. With support, they can travel to familiar places, and they can take care of themselves, their safety, and their health [3,19,22]. A person with a severe level of ID (making up 3.5% of ID) needs extensive support for daily activities. Language and capacity for the acquisition of academic skills are limited. Basic self-care skills may be acquired with intensive training, and motor impairments occur frequently. Individuals with a profound level of ID (making up 1.5% of ID) need intensive support for every aspect of daily routines. Their communication skills are limited, and motor or sensory impairments are frequent [3].

Intellectual functioning is more a continuum of abilities than a categorical variable, similar to AB. Borderline intellectual functioning (BIF) is at the border between normal intellectual functioning and intellectual disability. The intelligence quotient (IQ) is between 70 and 85 points. BIF is not a diagnosis in DSM or ICD, but it includes a heterogeneous group of specific neurodevelopmental syndromes, disorders, and extreme variations of normality [23]. These individuals do not meet the criteria for Disorders of Intellectual Development (DID). Their adaptive behavior varies, and some people with BIF can manage several areas of life without a need for support. Nevertheless, due to their impairments in cognitive and executive functions, AB, academic and social skills, and more mental health problems [24], their outcome is dependent on education, social connections, and personality [24]. People with BIF cannot gain access to support and services that are given to persons with DID, which makes their success in society difficult [25].

Institutionalization is the most common societal intervention for orphaned, abandoned, or maltreated children throughout the world [26]. In most cases, the reasons for placing children in institutional care are poverty and maltreatment, along with the hope to improve access to health care and education [27]. Children with disabilities are over-represented in institutions worldwide; however, the exact number of children in institutions, in general, but also specifically with disabilities or ID, is unknown [27,28]. Institutional care is also associated with increased rates of mental health problems. It is estimated that 50% of children have mental disorders [11]. Children with a history of institutional care/foster care show a higher rate of psychiatric symptoms in youth, including depression, anxiety, somatization, dissociation, and the symptom dimensions of posttraumatic stress disorder, while the impact of institutional care is more deleterious when compared to foster care [10,29,30]. Institutionalized children usually have a variety of genetic, pre-, peri- and postnatal risk factors. These risk factors are often associated with lower intellectual functioning [31]. More challenged children have a lower chance to be adopted and raised in foster families, and they stay longer in institutional care. All children in institutions are vulnerable [32] but children with disabilities are at higher risk of maltreatment [27,28,33], especially children with physical impairments are at risk of sexual abuse [34]. In recent years, several studies focused on AB in children or adolescents with maltreatment or placed in institutional care have been published. In the study of Viezel, Lowell and Davis [7], the authors have shown that neglected children had lower scores of AB in all domains (Communication, Daily Living Skills, Socialization) than abused children or children in the control group, and abused children had lower scores than the control group [7,35]. Deprivation is associated with lower cognitive abilities, especially verbal aspects [29,36,37], and poor social skills. Mediating, also moderating function between adversity and social problems is a disruption of reward processing [38,39].

A specific factor influencing AB of children in institutional care is a risk of cumulative lifetime traumatization, ongoing maltreatment in an institution, or institution-specific adverse effects, that may result in psychosocial deficits as a consequence of the institution’s care [30]. That includes frequent changing of caregivers, numbers of children in group care, presence of children with disability in the same group, not enough attention for an individual’s needs, separation from other social environments (e.g., allocated education group at living place), and lack of potential supporting systems (hobby, sports, peer groups, community life) [30,40]. Together with early life adverse circumstances, children in institutional care are at risk of worse outcomes in adulthood [40]. We assume that these factors also have a negative effect on adaptive behavior.

Intellectual disabilities are another significant predictor of mental health problems in children in institutional care [11,41,42]. One of the well-documented differences compared to children from families in the literature is the lower opportunity to create a secure attachment with a consistent caregiver [43,44]. Secure attachment is an important factor for individual resilience and positive adaptation [45]. Children with reactive attachment disorder and/or disinhibited social engagement disorder have significantly different abilities in the socialization and motor domain [46]. Becker-Weidman [47] reports a significant difference in the chronological and developmental age of children in foster care with a reactive relationship disorder. The most delayed area was communication (according to VABS-II). In addition to predominantly insecure attachment and difficulty recognizing emotions, these children are generally more lagging in cognitive development [43,44]. Furthermore, we can assume that cognitive abilities mediate the relationship between neglect, deprivation, and AB.

The aim of this study is to analyze the adaptive skills of a sample of children with intellectual disabilities in institutional care in Slovakia. The analysis reflects differences in adaptive domains related to intellectual functioning, gender, length of stay in institutional care, and type of institutional care. In the prediction model, we analyze the relationship of specific factors related to institutional care and pre-, peri- and postnatal risk factors in the development of AB. We included the level of intellectual functioning in the model also due to the assumed associations with other predictors that are specific for the children in institutional care. The age at institutionalization and length of stay may be indirectly linked with the severity of the ID and associated medical (somatic) conditions that have an impact on the AB [48]. In addition, some of the risk factors that we analyzed are also associated with the level of ID [31].

## 2. Materials and Methods

### 2.1. Sample

Our sample consisted of 197 children aged 5.3–18.8 years (mean = 12.8, SD = 2.97), of that 50% (*n* = 98) were boys. The inclusive criterion was intellect lower than IQ 85. In our sample, 16.8% of children had borderline IQ (84–70), and the remaining children had a diagnosed DID by ICD-10 criteria: of that, 56.8% were mild, 19.8% moderate, and 6.6% severe. Children with a profound level were excluded from further analysis due to their low count (2). All children were in institutional care in various forms (14.2% in professional foster care, 62.4% in group-based care, 18.3% in group-based care for children with disabilities, 5.1% in group-based for children with mental disorders). Children were placed in institutional care most often in preschool and younger school age (range 0–17 years old, mean 6.7 years), with a severe level of ID 3–4 years earlier than others. The mean length of stay in institutional care was 6.1 years (SD = 4.4), and the duration was the longest in children with a severe level of ID (M = 10.23 years, SD = 3.32 years). The ethnic composition of the sample was 83% Slovak, 14% Roma, and 3% Hungarian.

IQ of the children was examined for estimation of the child’s educational needs. IQ testing was performed by trained psychologists—diagnosticians from educational and psychological counseling institutions for children with special needs. Intellect was examined using standardized methods available in Slovakia (WISC-III, SON-R, S-B IV, K-ABC, Raven progressive matrices).

In the sample, children were diagnosed with the following co-occurring conditions: 4.6% (9) autism spectrum disorder (ASD), 3.6% (6) Down syndrome, 19.1% (18) cerebral palsy, 26.4% (52) communication disorders, 39.1% (77) conduct disorder, 16.2% (32) attention deficit and hyperactivity disorders (ADHD), 1.5% (3) SLD (specific learning disorders), 7.1% (14) anxiety disorder, 8.6% (17) depressive disorder, 4.1% (8) hearing impairment (different levels), 8.6% (17) visual impairment (different levels), and 10.2% (13) epilepsy. Characteristics of the sample are shown in Table 1.

### 2.2. Methods

Vineland Adaptive Behavior Scales, Third Edition (VABS-3) is a standardized measure of adaptive behavior [12]. We used the Slovak research version of the Comprehensive Form for parents/caregivers that was filled in by staff (caregivers) at children’s homes. VABS-3 assesses three domains of AB—Communication, Daily Living Skills, Socialization, and global AB Composite score ABC. Each domain includes 3 subdomains.

Communication domain: Receptive—attending and understanding and responding appropriately; Expressive—using words and sentences to express oneself verbally to others; Written—using reading and writing skills.

Daily Living Skills domain: Personal—self-sufficiency in such areas as eating, dressing, washing, hygiene, and health care; Domestic—performing household tasks such as cleaning up after oneself, chores, and food preparation; Community—functioning in the world outside the home, including safety, using money, travel, rights, and responsibilities, practically using numeric concepts—time, dates, and money.

Socialization domain: Interpersonal relationship—responding and relating to others, including friendship, caring, social appropriateness, and conversation; Play/Leisure—engaging in play and fun activities with others; Coping skills—demonstrating behavior and emotional control in different situations involving others.

Higher scores in domains and subdomains indicate better adaptive behaviors. Slovak normative data are not available; therefore, American normative data were used for the transformation of raw data to a standard score.

Anamnestic questionnaire. Caregivers (staff) in institutional care or professional foster parents filled in an anamnestic questionnaire regarding demographic information, risk factors in development, and information about institutional care—length, and type of institutional care (professional foster care vs. group-based care). Information on risk factors was obtained from children’s documentation. Due to the large heterogeneity of data in the quality of information, we decided to merge some variables into umbrella variables. An explanation of individual risk factors is summarized below.

Substance use during pregnancy—alcohol and opioid use during pregnancy. It is not necessary for the child to have symptoms of neonatal abstinence syndrome (NAS) or fetal alcohol syndrome (FAS).

Neonatal infection—in utero infection such as syphilis, toxoplasmosis, CMV, hepatitis B, etc.

Prematurity—we did not differentiate the degree of prematurity. We considered all children born before the 38th week of gestation to be premature.

Labor complications—information in health documentation about any complication during labor, such as fetal distress, perinatal asphyxia, fetal injury, umbilical cord problems, etc.

Neonatal difficulties—every serious somatic complication after birth in the first 28 days, e.g., necrotizing enterocolitis, neonatal pneumonia, sepsis, hemorrhagic disease, and congenital heart defects.

Neglect of early health care (Neglect of EHC)—omitting regular preventive examinations and professional examinations in the first three years of a child’s life, that would lead to early identification of somatic and/or developmental problems. Omission of recommended treatment procedures—e.g., rehabilitation.

Maltreatment—includes all types of physical and/or emotional maltreatment, sexual abuse, and neglect in the child’s past.

### 2.3. Data Analysis

Statistical analysis was performed with SPSS Version 22 Statistics for Windows, and Jamovi [49]. Normality tests were performed using histogram, Q-Q plot, Skewness, and Kurtosis. A one-way ANOVA and chi-square analysis were used to confirm that there were no differences between the four groups with respect to age and gender. Basic data analysis included Pearson correlations (VABS scores and age, VABS scores and Length of stay), and Independent Samples *t*-test (VABS scores and gender). One-way ANOVA (Fisher’s statistics, Welch statistics, Games–Howell post hoc test) was conducted to examine differences in adaptive behavior between groups. A paired *t*-test was used for the analysis of differences between AB domains within groups. Hierarchical regression models in 4 steps were used to examine if independent variables (Level of ID, Gender, Length of stay, Type of institutional care, Substance use during pregnancy, Neonatal Difficulties, Neglect EHC, Prematurity, Labor complications, Neonatal infection, and Maltreatment) could be relevant predictors for AB (standardized ACB composite score of VABS).

In the analyses, we used V-scores (M = 15, SD = 3) for the subdomains and standard scores (M = 100, SD = 15) for the AB domains and the ABC composite score. Data were expressed as means (M) ± standard deviation (SD). A value of *p* < 0.05 was considered statistically significant.

## 3. Results

A one-way ANOVA (Fisher’s statistics) has shown no significant differences in age in the four groups F(3,193) = 0.71, *p* = 0.548, η2 = 0.011: BIF (M = 13.4, SD = 2.91), Mild ID (M = 12.6, SD = 2.87), Moderate ID (12.8, SD = 3.3), Severe ID (M = 13.1, SD = 2.96). A chi-square test of independence showed that there was no significant association between Gender and Level of ID, X^2^ = (3,197) = 2.74, *p* = 0.434, Cramer’s V = 0.118.

### 3.1. Comparison of Adaptive Behavior between Levels of ID

One-way ANOVA (Welch statistics, Games–Howell post hoc test) showed that there was a statistically significant difference in ABC score and all AB domains between groups (Level of ID) except for BIF and Mild ID groups in the Socialization domain (*p* = 0.265). With the increasing severity of ID, a progressive decline in AB standard scores was observed. Overall, the highest scores were achieved in all dimensions by children with BIF, and within the dimension, all children achieved the highest scores in Daily Living Skills (Table 2). Mean differences and post hoc analysis are reported in Appendix A Table A1.

We found very similar results when evaluating the subdomains (Table 3). Based on the results of the post hoc analysis (Games–Howell post hoc test), the groups differed significantly in all subdomains except for children with BIF and Mild ID. They differed significantly only in the subdomains Daily Living Skills—Personal (*p* = 0.007), Daily Living Skills—Community (*p* < 0.001), and Communication—Written (*p* < 0.001). In the other subdomains, children from these two groups achieved very similar results: Socialization—Interpersonal (*p* = 0.558), Socialization—Play/Leisure (*p* = 0.292), Socialization—Coping Skills (*p* = 0.760), Daily Living Skills—Domestic (*p* = 0.319), Communication—Receptive (*p* = 0.162), and Communication—Expressive (*p* = 0.200). Descriptive statistics of each subdomain, mean differences, and post hoc analysis are reported in Appendix A Table A2 and Table A3.

### 3.2. Profile of Adaptive Behavior for Each Level of ID

**Figure 1:**
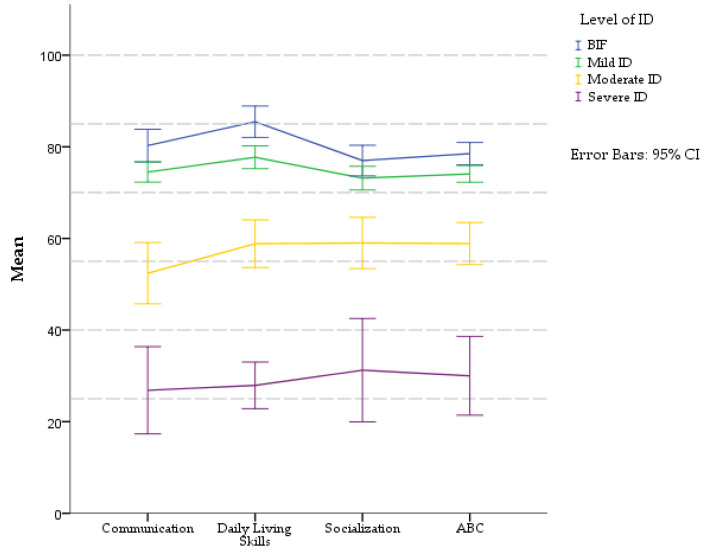
Profiles of AB domains (VABS-3) in different levels of ID. Standard scores: M = 100, SD = 15.

A profile of adaptive domains was analyzed for each level of ID (Figure 1). The paired-sample *t*-test was used for pairwise comparison of standard scores in domains of AB on each level of ID (in Appendix A Table A5).

The profile of AB in the BIF group was characterized by Daily Living Skills > Communication > Socialization. Children scored better in the domain Daily Living Skills (M = 85.4, SD = 9.67), as compared to Communication (M = 80.27, SD = 10.1). This difference was statistically significant, t(32) = −3.157, *p* = 0.003. The poorest adaptive abilities were observed in the Socialization domain (M = 77, SD = 9.43), in comparison to Daily Living Skills, and this difference was significant t(32) = 5.530, *p* < 0.001.

A mild level of ID displayed similar results as BIF with the best abilities in Daily Living Skills domain (M = 77.7, SD = 13.31), which was significantly different from the Communication (M = 74.48, SD = 11.76), t(111) = −3.60, *p* < 0.001. The score was lowest in the Socialization domain (M = 73.16, SD = 13.75), and this difference was statistically significant when compared to the Daily Living Skills t(111) = 4.75, *p* < 0.001.

The profile of AB in the group of children with a moderate level of ID was Socialization > Daily Living Skills > Communication. Differences between domains were statistically significant in: Communication (M = 52.41, SD = 20.61) and Daily Living Skills (M = 58.85, SD = 16.1), t(38) = −3.313, *p* = 0.002, and Communication vs. Socialization (M = 59, SD = 17.27) t(38) = −2.87, *p* = 0.007. Children with a severe level of ID had similar profiles as the group with Moderate ID: Socialization (M = 31.23, SD = 18.68) > Daily Living Skills (M = 27.92, SD = 8.4) > Communication (26.85, SD = 15.74). Differences between Communication and Socialization were statistically significant (t(12) = −3.156, *p* = 0.008). Other domains displayed no significant differences.

**Figure 2:**
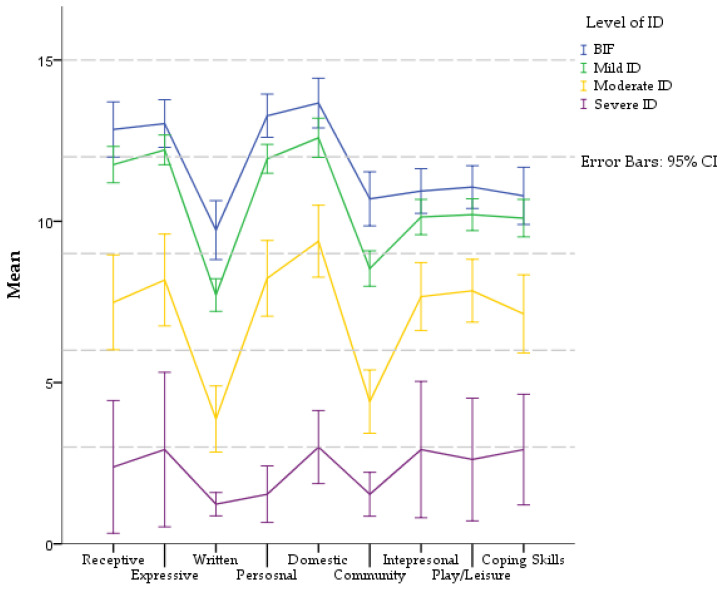
Profiles of AB (subdomains VABS-3) in different levels of ID. Communication domain includes the Receptive, Expressive, and Written subdomains. Daily Living Skills include the Personal, Domestic, and Community subdomains. Socialization domain includes the Interpersonal, Play/Leisure, and Coping Skills subdomains. All subdomains are in V-score (M = 15, SD = 3).

To provide more detailed information about the AB of children in institutional care, Figure 2 represents profiles for each level of ID in VABS-3 subdomains. Complete information about pairwise *t*-tests is shown in Appendix A (Table A4).

All groups had the same profile of competencies in the Communication domain: Expressive > Receptive > Written. However, the significance differs in individual levels of ID. In BIF and Moderate ID groups, no significant difference between receptive and expressive communication was found. None of the communication skills in the Severe ID group differed significantly from each other. The differences between other communication subdomains in each level of ID were significant.

In the Daily Living Skills domain, the profile of competencies was the same in all groups: Domestic **>** Personal > Community. Differences between skills were significant in all groups, except in BIF, where the difference between Domestic and Personal was not significant.

The Socialization domain had a uniform profile in each level of ID. In group BIF, Mild ID and Moderate ID was profile same: Play and Leisure > Interpersonal > Coping skills. Differences between each skill did not differ significantly except in the Moderate ID group, where Play and Leisure scored higher than Coping skills. Children with Severe ID had a different profile: Interpersonal = Coping skills > Play and Leisure. They had developed these skills at a very low level.

### 3.3. Gender Differences in Adaptive Behavior

Gender was related to several AB domain scores (Table 4). The independent *t*-test showed that girls overperformed boys in Socialization and Daily Living Skills domains and ABC score, with the biggest difference in Daily Living Skills—mean difference of 9.86, CI95% [4.53, 15.2]. Results indicate non-significant differences between girls and boys in Communication. Girls overperformed boys also in all subdomains except for Expressive. The effect was biggest in the Domestic subdomain with a mean difference of 2.31 CI95% [1.21, 3.40].

### 3.4. Hierarchical Multiple Regression Model

A hierarchical multiple regression model was performed to examine the relationship between 11 independent variables (Level of ID, Gender, Type of institutional care, Length of stay in institutional care, Substance use during pregnancy, Neonatal Difficulties, Neglect EHC, Prematurity, Labor complications, Neonatal infection, Maltreatment) and AB (standardized ACB composite score of VABS-3). Preliminary analyses were conducted to ensure no violation of the assumptions of normality, linearity, and homoscedasticity.

**Figure 3:**
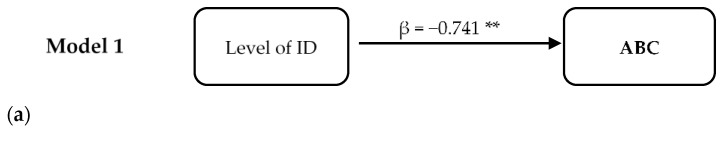

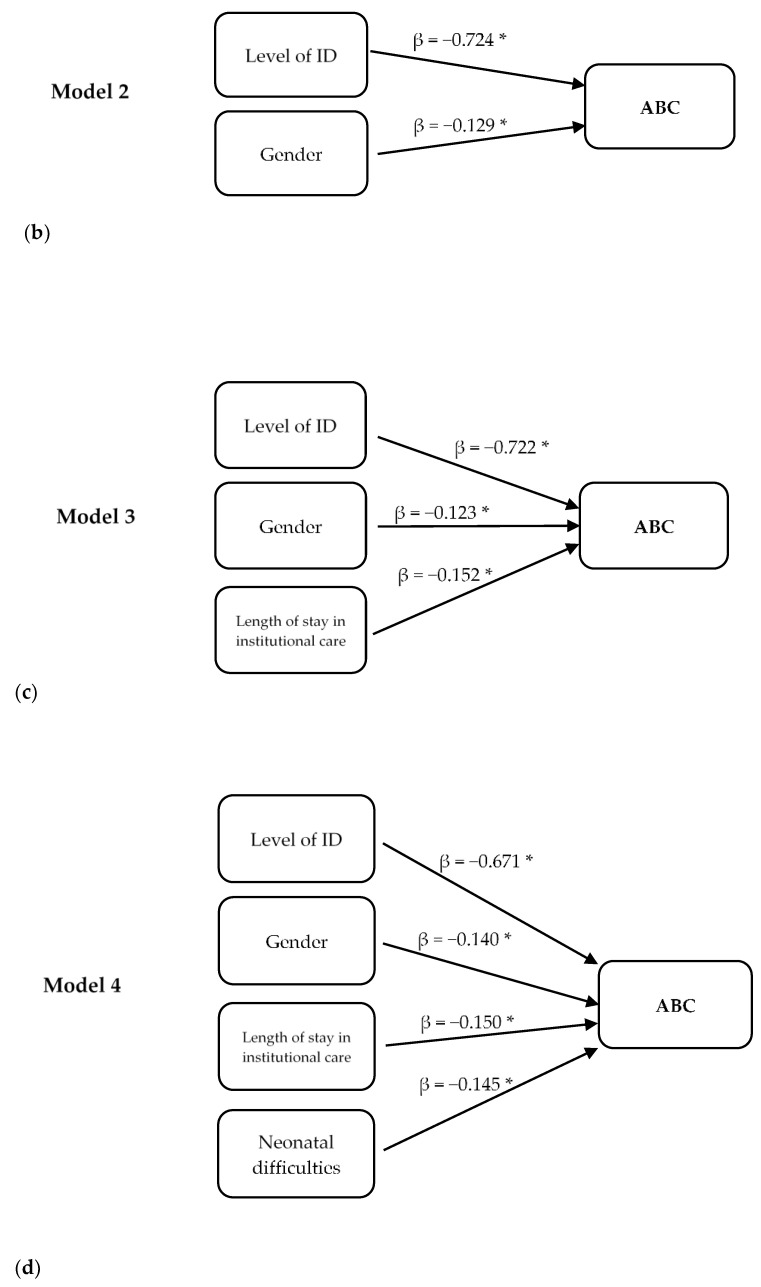
Hierarchical regression models for predicting ABC scores. Significant predictors were in four steps of regression: (**a**) Level of ID, (**b**) Level of ID and Gender of children, (**c**) Level of ID, Gender, and factors related to institutional care—Length of stay and Type of institutional care, (**d**) Level of ID, Gender, factors related to institutional care—Length of stay, Type of institutional care, and risk factors in development—Neonatal difficulties. * *p* < 0.05, ** *p* < 0.01.

Figure 3 represents four hierarchical models.

In the first step, the level of ID accounted for 54.8% of the variance. The model was statistically significant F(1,142) = 172.44, *p* < 0.001, *R^2^* = 0.548.

In the second step, Gender accounted for an additional 2% of variance. The model was statistically significant F(2,141) = 91.53, *p* < 0.001, R^2^ = 0.565, R^2^_Adjusted_ = 0.559). Both Level of ID (*p* < 0.001) and Gender (*p* = 0.022) were significantly associated with AB.

After entry of Length of stay in institutional care and Type of institutional care at Step 3, the total variance explained by the model was 59.9%, F(4,139) = 51.98, *p* < 0.001, R^2^ = 0.599, R^2^_Adjusted_ = 0.588. Only the Level of ID (*p* < 0.001), Gender (*p* = 0.025), and Length of stay (*p* = 0.007) significantly contributed to the model.

After the entry of risk factors in the development (Neonatal Difficulties, Neglect of early health care, Prematurity, Labor complications, Neonatal infection, Substance use during pregnancy, Maltreatment), the total variance explained by the model was 63.6%, F(11,132) = 20.935, *p* < 0.001, R^2^ = 0.636, R^2^_Adjusted_ = 0.605.

In the final model, 4 out of 11 predictor variables were statistically significant, including the Level of ID (*p* < 0.001), Gender (*p* = 0.011), Neonatal Difficulties (*p* = 0.029), and Length of stay in institutional care (*p* = 0.009).

## 4. Discussion

The aim of the study was to analyze AB in children with ID and BIF in institutional care. We focused on a specific vulnerable group of children who depend on the support of the social system much more than children who have ID and grow up in families.

### 4.1. Differences of AB in Severity Levels of ID and BIF Groups

We analyzed AB in the different levels of ID and BIF groups. In our sample, a significant difference was present in the domains of AB and in ABC scores, which supports previous findings of decreasing AB with decreasing levels of ID [1,24,25,50,51]. BIF and Mild ID groups differed in Communication and Daily Living Skills but not in Socialization. Compared to normative data of ABC composite score and AB domains, the BIF and Mild ID group scored in the range of 1 to 2 SD below the mean [12]. Significant limitations in AB are one of the criteria for a diagnosis of ID, which means an AB score that is “approximately two standard deviations below the mean in one of the three adaptive skills areas” [2].

Children in our sample were diagnosed according to ICD-10 criteria (in that time being the official classification system), where the assessment of adaptive behavior was not one of the essential criteria of ID. The severity level of ID was determined mainly by the result of the IQ test. If the ICD-11 criteria were applied, it is possible that some of the children who are in the Mild ID group would no longer meet these criteria [52]. Another explanation of the small differences between BIF and Mild ID groups may be the relationship between AB and IQ itself. This relationship, however, tended to decrease as IQ increased [51]. Not everyone with ID must also present significant deficits in AB, and conversely, those with significant deficits in AB may not also have ID (e.g., children with ASD and average intellect) [53]. Next, stronger correlations between intelligence and adaptive behavior are expected in younger children, especially children in the preschool period [51]. The mean age of our sample was 12.8 years.

In a more detailed analysis of the results in the BIF and Mild ID groups, a significant difference was observed only in some subdomains. The Written subdomain reflects the quality of reading, writing, and working with information. These skills are closely linked to cognitive processes and require more executive control than receptive and expressive communication. We expected the Mild ID group to have a weaker cognitive capacity to solve more complex tasks than the BIF group. However, the quality of reading and writing also depends on the method of education and stimulation. In Slovakia, most children with Mild ID are educated in schools for children with special needs, separated from other children (without ID) [54]. These schools are not a tool for temporary support but represent a permanent separate educational path, that has an extremely negative impact on some groups of children and limits their opportunities to achieve a complete primary education [54,55].

Despite the similar profile of AB in the BIF and Mild ID groups, we found significant differences in the Personal subdomain that reflects self-care, such as feeding, dressing, hygiene habits, and health care. Children with BIF scored in the range up to 1 SD below the mean and overperformed the Mild ID group. Similarly, the BIF group scored better in the subdomain Community, which reflects skills to function outside the home, e.g., using money, traveling, respecting others’ rights, etc.

Not all children with BIF have problems with adaptive behavior. However, both BIF and Mild ID groups are at greater risk of social isolation. Problems in contact with peers are very common, and social challenges are similar for both groups [24]. Although the cognitive potential of children with BIF is better, they seem to be exposed to greater psychosocial issues than children with Mild ID. Children with BIF have more mental health problems and more complicated family backgrounds [56]. These factors may contribute to the explanation of the similarity of the quality of Socialization in both groups, which ranged from 1 to 2 SD below the average.

Another possible explanation for the small differences between BIF and Mild ID is the specificity of children in institutional care. The care for these children was neglected during their sensitive developmental period. The conflicting relationship between the caregivers and the children is in negative relation to children’s adaptive skills [9]. Maltreatment has long-term consequences for the children’s development, also for AB [7,35]. Children with BIF and Mild ID were placed in institutional care at the time (M = 7.5 years) when their primary education begins (after reaching 6 years). It has been shown that neglect and a low-stimulating environment can lead to problems in the assessment of intellect, also in making decisions about special needs education. Children with BIF also have poorer access to the support system than children with ID, so sometimes conclusions of assessment can be related to access to this help [25].

### 4.2. Profiles of AB in Severity Levels of ID and BIF Groups

BIF and Mild ID groups had similar profiles of adaptive domains with the best performance in Daily Living Skills, followed by Communication, and weakest in the Socialization domain. Daily Living Skills were approximately 1 SD below the mean in the BIF group and 1.5 SD below the mean in the Mild ID group. Scores in the Domestic subdomain were highest in both the Mild ID and BIF groups. In our sample, 15% of children with BIF and 18% with Mild ID were placed in professional foster care, which is closest to family life. There were 79% of children with BIF and 70% with Mild ID placed in group-based care. Children in group-based care live in a household with a maximum of 8 children and are cared for by 3–4 alternating caregivers. Children in group-based care are involved in shopping, cleaning up after themselves, chores, food preparation, and cooking, helping younger children. The weakest area in the Daily Living Skills is Community in all groups in our sample. The mean score in BIF was −1 SD, and in the Mild ID group, it was below −2 SD. Problems include difficulties searching for information online, using communication technologies, time and date orientation, money, travel, and traffic orientation, safety behavior, etc. We assume that the poorer score in this subdomain is due to institutional care, which limits children’s ability to function independently in the world outside the home. Overall, we can consider Daily Living Skills as a strength of children in institutional care [57].

On the contrary, Socialization is manifested in the BIF and Mild ID groups as the weakest area; their average values were between 1 and 2 SD below the mean. Socialization may have been impaired due to maltreatment or early adverse experience. Maltreatment can impact children’s coping skills, attachment, social functioning, and psychopathology. The quality of socialization is closely connected with challenging behavior and mental health problems [38,39,58,59]. Intellectual disabilities are a significant predictor of mental health problems in children in institutional care [11,42]. A stay in institutional care can also have a negative effect on the quality of relationships [43,44].

In the Communication domains, the Written subdomain was the weakest for both groups and in all severity levels of ID. Reading and writing are very complex cognitive processes sensitive to a decline in cognitive abilities. For children with ID, however, reading can be an area of relative weakness but there is evidence that it can also be a strength [60].

A different pattern was observed in the Moderate and Severe ID groups. They reached the best scores in the Socialization domain, followed by the Daily Living Skills, and the poorest performance was in the Communication domain. Moderate and Severe ID are often associated with genetic factors and somatic, motor, and sensory impairments that negatively impact AB. In the Moderate ID group, Socialization was at a similar level as Daily Living Skills, i.e., 3 SD below the average. This group had a significant decline in the Written subdomain in the Communication domain, similar to all the other groups. In the Severe ID group, the decrease in AB in all domains is 5 SD below the mean. Persons with Severe ID typically display impairment in motor domains and significant deficits in communication [2], which we can also see in our sample.

When the profile of AB is analyzed, the co-occurring disorders and neonatal risk factors should be considered, as they may have a different impact on cognitive functions and may lead to varying levels of intellectual disability [61,62,63,64,65,66]. Furthermore, the stability of the AB profile for individuals is different. For example, with the Fragile X chromosome, there may be a decrease in AB over time. However, females and males have different trajectories [61]. On the contrary, at Prader Willi syndrome, the AB remains stable over time [67]. Our sample included a relatively large age range (5–18 years) and is heterogeneous in terms of the presence of co-occurring disorders (Down syndrome, ASD, SLD, ADHD) or syndromes (cerebral palsy). Therefore, the AB profiles for a single ID level represent a certain simplification of our result.

### 4.3. Gender Differences in AB

We observed gender differences in Daily Living Skills, Socialization and ABC score by 1 SD. In all these areas, the girls overperformed the boys. Some studies that include typically developing children and children with neurodevelopmental disorders also point to gender differences in adaptive behavior [17,57,68,69,70]. Differences are often in Communication domains, where girls, both in the general population and with disorders, have better results [17]. In our group, girls scored higher in Communication, but the difference was not significant. Significant differences were observed only in the subdomains Receptive and Written.

Our sample’s most significant gender differences were in Daily Living Skills and its subdomain Domestic. It may also be related to the cultural context, different expectations, and attitudes toward raising boys and girls. We assume that girls are more involved in the household, such as cooking, cleaning, etc. [18]. We presume that institutional care is likely to copy social trends.

In a longitudinal study of Japanese children from the general population, gender differences were observed in the Daily Living Skills and Socialization domains, especially in children with lower intelligence; on the contrary, gender differences were not observed in children with average intelligence. These differences persisted over time [17].

The VABS-3 instrument does not consider gender differences [15]. The different trajectories for girls and boys suggest that assessing adaptive behavior requires consideration of the child’s gender. It may play an essential role in the early identification of children with deficits (e.g., ASD) [17].

### 4.4. Predictors of AB

The level of ID accounted for 54.8% of the variance in AB. Correspondingly with our assumptions, it was the most significant predictor. Gender accounted for an additional 2% of variance. In the next model, we analyzed specific predictors of AB related to institutional care. The type of care, i.e., comparison of professional foster care (close to life in a family with the primary caregiver) with group-based care (in which the child grows up in a group of several children without a primary caregiver) was not a significant predictor. Only the Length of stay in institutional care was a significant predictor for AB. This factor accounted for an additional 3.1% of variance of the AB. The Length of stay in institutional care was weakly negatively correlated with the quality of AB, similar to the results of a previous study with infants and toddlers in institutional care [57]. The longest stay was observed in children with Severe ID (mean ten years,) and at the same time, they were placed in institutional care at the youngest age (before three years of their age) compared to other groups (range of 6–7 years old). Often, these are children who also have other somatic complications and diseases. Therefore, there is only a small probability that these children will return to their biological families, or that they will be adopted. In a meta-analysis by Goemans and van Geel and Vedder [71], it was pointed out that children do not improve their AB while in foster care. Some studies have shown that a long-term stay in foster care had a negative impact on AB [71]. Institutional care can only partially “catchup” with lagging in development. The specifics of institutional care also negatively affect the development of a child [72]. Difficulties in developing a secure attachment, frequent changes of caregivers, and a large group of children with whom they grow up, can negatively affect development and bring specific adverse experiences to the child [30,40,57]. The type of institutional care was not a significant factor in predicting AB in our sample. Therefore, the assumption that staying in professional foster care compared to group-based care could have a better effect on AB, was not confirmed. Our study did not monitor how long the child was in this type of care. During the stay, children often alternate the types of care, and professional parents can also alternate. The stability of the environment in foster care is one of the most critical factors that can affect a child’s development and well-being [42].

Risks in development (Neonatal Difficulties, Neglect of early health care, Prematurity, Labor complications, Neonatal infection, Substance use during pregnancy, Maltreatment) accounted for an additional 3.4% variance of AB in our sample. Risk factors better predicted the level of intellect than AB alone [30]. Circumstances during childbirth or just after birth are often associated with an increased risk for neurodevelopmental difficulties or cognitive deficits [61,65,73,74,75]. However, the factors we followed are not very specific for AB outcome and we assume only their indirect effect on AB.

In conclusion, the quality of AB results from several factors that influence the child’s development. We were able to explain 63% of the variance by combining various factors related to cognitive abilities, the quality of institutional care, and risk factors in the child’s development.

### 4.5. Implications and Limits of the Study

Our results highlight the difficulties of children with BIF in AB. BIF and Mild ID groups are close in terms of AB and both groups have similar challenges. This result emphasizes the need to provide systematic help even to children who do not meet the diagnostic criteria for ID. These are the groups of children who have the best perspective on becoming independent after leaving institutionalized care. The transition to independence in adulthood is often difficult, many children do not have sufficient skills [76]. The area that requires special attention is the functioning outside the home, where the Mild ID and BIF groups had the greatest deficits in their abilities.

Another important result of our study is the gender differences in AB in children with ID and BIF. This supports the need to have adequate gender-specific AB assessment tools [17].

The results of this study can be applied in social prevention, gynecology, and primary pediatrics. Early identification of at-risk mothers and their children, and intervention might reduce the negative impact on children’s development. Prevention should also focus on families of children with ID and BIF, that are exposed to complex problems [77]. Problems in the family context increase the likelihood of adverse childhood experiences and this in turn increases the likelihood of the child being placed in institutional care [78]. Therefore, it is important to focus on the high-risk children with pre-, peri- and postnatal difficulties and provide them with early health and social intervention, which has the potential to reduce the risk of adverse circumstances in the child’s development. In addition to social support, for example, support for parenting skills can lead to better health care for the child.

Identifying the at-risk mothers could help to reduce the harm for the mother, but also to her child [79]. Even though there are no consistent findings regarding the impact of a parent’s addiction on a child’s outcome, an intervention program focused on the relationship between a child and a parent has the potential to improve parents’ sensitivity to the child, the ability to apply appropriate parenting strategies that have a long-term effect on the child’s development [80,81]. By supporting the parents and the at-risk mothers, we can prevent the placement of children in institutional care.

Our study has several limitations that reduce the possibility of generalizing our results. The splitting of the sample into groups according to the level of ID was only based on anamnestic data. As we have seen, the results of the BIF and Mild ID groups were very close, which could be due to the inaccurate diagnosis of intellectual disorders. We were missing several pieces of information about the development, health, and social background of the children that would have helped to refine our analyses. For example, prematurity has different degrees that we could not distinguish due to the lack of detailed data and thus there might have been interfering health complications with an impact on AB. Many children in institutional care lack comprehensive information about their health status, also about their psychosocial development. The unavailability of this information is linked to the reasons why children end up in institutional care.

We did not consider the presence of comorbidity, which can affect the outcome of AB. For example, it has been shown that in ASD or ADHD, the presence of these disorders reduces the quality of AB. In the future, it would be appropriate to analyze AB not only according to the level of ID but also to work with specific groups, e.g., children with Down syndrome, ASD, ADHD, etc. Analysis of such profiles could provide more specific information about the needs of these children. Our results have a cross-sectional design, which limits making specific predictions. Prospective monitoring of children in institutional care would make it possible to better identify the factors that have an impact on AB and thus apply appropriate interventions.

In the analyses, we worked with a standard score that is derived from American normative data. There is no information about the Slovak population against which it would be possible to compare these children. Distinct environments have different expectations for age-related adaptive abilities [12,82,83]; therefore, we need adequate normative data to assess AB. Research findings on Slovak children or adolescents are still marginal [52].

We also needed to consider the Slovak context, such as the specifics of Slovak institutional care (e.g., organization of care), the poor quality of assessment tools (availability and out of date) used in the diagnostics process of ID, specifics in the education of children with disabilities (e.g., limited inclusiveness).

## 5. Conclusions

This study provided cross-sectional information on the adaptive behavior of children with ID and BIF in institutional care. Profiles for the level of ID and BIF were described. We have identified gender differences, which are important for assessment and practices. The strongest predictor of AB was the level of ID. Risk factors in development and factor associated with institutional care were not very specific for AB outcome and we assume only their indirect effect on AB.

## Figures and Tables

**Table 1:** Profile of the study sample.

	BIF N = 33 (16.8%)	Mild N = 112 (56.8%)	Moderate N = 39 (19.8%)	Severe N = 13 (6.6%)	Sample Total N = 197 (100.0%)
Gender					
Boys	19 (57.6)	55 (49.1)	20 (51.3)	4 (30.8)	98 (49.7)
Girls	14 (42.4)	57 (50.9)	19 (48.7)	9 (69.2)	99 (50.3)
Age (in years)					
Mean (SD)	13.4 (2.9)	12.6 (2.9)	12.8 (3.3)	13.1 (3.0)	12.8 (3.0)
Range	6.8–17.9	5.3–18.8	6.1–17.7	7.7–18.7	5.3–18.8
Type of institutional care					
Professional foster parents	5 (15.2)	20 (17.9)	3 (7.7)	-	28 (14.2)
GBC	26 (78.8)	78 (69.6)	19 (48.7)	-	123 (62.4)
GBC for children with disability	2 (6.1)	11 (9.8)	11 (28.2)	12 (92.3)	36 (18.3)
GBC for children with mental disorders	-	3 (2.7)	6 (15.4)	1 (7.7)	10 (5.1)
Age of institutionalization					
Mean (SD)	7.3 (3.7)	7.1 (4.2)	6.4 (4.9)	2.8 (3.4)	6.7 (4.3)
Range	1.1–17.6	0.8–15.7	0.1–16.1	0–11	0.8–17.6
Length of stay in institutional care (in years)					
Mean (SD)	6.1 (3.9)	5.5 (4.1)	6.3 (5.2)	10.2 (3.3)	6.1 (4.4)
Range	0.0–12.0	0.4–16.2	0.3–17.0	4.0–15.0	0.0–17.0
Risk factors in the development *					
Substance use during pregnancy	7 (21)	28 (25)	6 (15)	1 (7.7)	42 (22.6)
Neonatal infection	1 (3.0)	5 (4.5)	8 (20.5)	3 (23.1)	17 (8.6)
Prematurity	5 (15.2)	14 (12.5)	10 (25.6)	7 (53.8)	36 (18.3)
Labor complications	3 (9.1)	6 (5.4)	9 (23.1)	2 (15.4)	20 (10.2)
Neonatal difficulties	3 (9.1)	11 (9.8)	12 (30.8)	5 (38.5)	31 (15.7)
Neglect EHC	9 (27.3)	35 (31.2)	16 (41.0)	-	60 (30.5)
Maltreatment	5 (15.2)	13 (11.6)	7 (17.9)	-	25 (12.7)
Not available	11 (33.3)	34 (30.3)	5 (12.8)	1 (7.6)	51 (25.8)

Note. BIF—borderline intellectual functioning; GBC—group-based care; Neglect EHC—neglect early health care. * 25.8% (51) of children did not have available information about risk factors in development.

**Table 2:** Comparison of Standard scores for AB domain in each level of ID.

							One-Way ANOVA			
	Levels of ID	N	M	SD	Min	Max	F_Welch_	Df1	Df2	*p*
Communication ^a^	BIF	33	80.3	10.07	53	105	54.7	3	42.4	<0.001
	Mild ID	112	74.5	11.76	30	107				
	Moderate ID	39	52.4	20.61	20	85				
	Severe ID	13	26.8	15.75	20	66				
Daily Living Skills ^a^	BIF	33	85.5	9.67	69	104	151.4	3	49.2	<0.001
	Mild ID	112	77.7	13.31	48	116				
	Moderate ID	39	58.8	16.06	26	95				
	Severe ID	13	27.9	8.40	20	50				
Socialization ^a^	BIF	33	77	9.43	56	98	30.3	3	43.6	<0.001
	Mild ID	112	73.2	13.75	29	103				
	Moderate ID	39	59	17.27	28	89				
	Severe ID	13	31.2	18.68	20	72				
ABC ^a^	BIF	33	78.5	6.89	68	99	58.2	3	43.0	<0.001
	Mild ID	112	74.1	9.73	45	107				
	Moderate ID	39	58.9	14.13	30	84				
	Severe ID	13	30	14.23	20	62				

Note. BIF—borderline intellectual functioning; ID—intellectual disability; ABC—composite score. ^a^ Standard score (M = 100, SD = 15).

**Table 3:** Group (Level of ID) differences between subdomains of AB.

	F_Welch_	Df1	Df2	*p*
Communication				
Receptive	42.9	3	43.6	<0.001
Expressive	34.2	3	41.8	<0.001
Written	212.1	3	73.8	<0.001
Daily Living Skills				
Personal	201.7	3	49.6	<0.001
Domestic	104.8	3	50.6	<0.001
Community	139.1	3	59.0	<0.001
Socialization				
Interpersonal	25.8	3	44.3	<0.001
Play/Leisure	32.9	3	44.1	<0.001
Coping Skills	31.7	3	45.2	<0.001

**Table 4:** Descriptive data for domains and subdomains of VABS-3 and Gender.

								*t*-test			
		Gender	N	M	SD	Min	Max	t	df	*p*	d
Domains	Communication ^a^	Girls	98	71.8	18.87	20	107	2.73	195	0.077	0.39
		Boys	99	64.1	20.57	20	96				
	Daily Living Skills ^a^	Girls	98	76.9	18.42	20	116	3.65	195	<0.001	0.52
		Boys	99	67.1	19.50	20	106				
	Socialization ^a^	Girls	98	73.0	15.77	20	100	3.77	195	<0.001	0.54
		Boys	99	63.5	19.46	20	103				
Composite score	ABC ^a^	Girls	98	72.9	14.99	20	107	3.54	195	<0.001	0.51
		Boys	99	64.9	16.52	21	90				
Communication	Receptive ^b^	Girls	98	11.29	4.03	1	18	2.64	195	0.009	0.38
		Boys	99	9.68	4.49	1	17				
	Expressive ^b^	Girls	98	11.44	3.74	1	17	1.74	195	0.084	0.25
		Boys	99	10.44	4.27	1	17				
	Written ^b^	Girls	98	7.57	3.55	1	16	2.81	195	0.006	0.40
		Boys	99	6.16	3.51	1	14				
Daily Living Skills	Personal ^b^	Girls	98	11.60	3.64	1	19	3.15	195	0.002	0.45
		Boys	99	9.89	3.99	1	16				
	Domestic ^b^	Girls	98	12.66	3.64	1	19	4.16	195	<0.001	0.59
		Boys	99	10.32	4.13	1	19				
	Community ^b^	Girls	98	8.43	3.61	1	18	3.06	195	0.002	0.44
		Boys	99	6.82	3.76	1	14				
Socialization	Interpersonal ^b^	Girls	98	10.29	3.04	1	17	4.05	195	<0.001	0.58
		Boys	99	8.33	3.68	1	17				
	Play/Leisure ^b^	Girls	98	10.08	2.99	1	16	2.98	195	0.003	0.43
		Boys	99	8.69	3.54	1	15				
	Coping Skills ^b^	Girls	98	10.05	3.33	1	17	3.44	195	<0.001	0.49
		Boys	99	8.26	3.93	1	16				

^a^ Standard score (M = 100, SD = 15). ^b^ V-score (M = 15, SD = 3).

## Data Availability

Data are available from the corresponding author upon reasonable request.

## Appendix A

**Table A1:** Post hoc comparisons of AB domain in different levels of ID.

	Comparison	Mean Difference	df	t	*p*_(Games-Howwel)_
Communication	BIF–Mild ID	5.79	60.1	2.79	0.035
	BIF–Moderate ID	27.9	57.1	7.46	<0.001
	BIF–Severe ID	53.4	16.0	11.35	<0.001
	Mild ID–Moderate ID	22.1	46.9	6.34	<0.001
	Mild ID–Severe ID	47.6	13.6	10.57	<0.001
	Moderate ID–Severe ID	25.6	26.9	4.67	<0.001
Daily Living Skills	BIF–Mild ID	7.77	71.3	3.70	0.002
	BIF–Moderate ID	26.6	63.7	8.67	<0.001
	BIF–Severe ID	57.6	25.2	20.3	<0.001
	Mild ID–Moderate ID	18.9	57.2	6.59	<0.001
	Mild ID–Severe ID	49.8	19.8	18.81	<0.001
	Moderate ID–Severe ID	30.9	40.2	8.91	<0.001
Socialization	BIF–Mild ID	3.84	76.0	1.83	0.265
	BIF–Moderate ID	18.0	60.6	5.60	<0.001
	BIF–Severe ID	45.8	14.5	8.42	<0.001
	Mild ID–Moderate ID	14.2	55.7	4.64	<0.001
	Mild ID–Severe ID	41.9	13.6	7.85	<0.001
	Moderate ID–Severe ID	27.8	19.3	4.73	<0.001
ABC	BIF–Mild ID	4.43	73.3	2.93	0.023
	BIF–Moderate ID	19.6	57.0	7.67	<0.001
	BIF–Severe ID	48.5	14.3	11.76	<0.001
	Mild ID–Moderate ID	15.2	51.1	6.23	<0.001
	Mild ID–Severe ID	44.1	13.3	10.88	<0.001
	Moderate ID–Severe ID	28.9	20.5	6.35	<0.001

**Table A2:** Descriptive of subdomains VABS-3 in each level of ID.

Levels of ID		N	Mean	SD	Minimum	Maximum
BIF	Receptive	33	12.85	2.41	6	18
	Expressive	33	13.3	2.8	8	17
	Written	33	9.73	2.58	6	15
	Personal	33	13.27	1.89	9	18
	Domestic	33	13.67	2.17	9	17
	Community	33	10.70	2.36	7	15
	Interpersonal	33	10.94	1.95	7	15
	Play/Leisure	33	11.6	1.87	6	14
	Coping Skills	33	10.79	2.50	5	16
Mild ID	Receptive	112	11.76	3.1	1	17
	Expressive	112	12.21	2.47	3	17
	Written	112	7.71	2.71	1	16
	Personal	112	11.94	2.39	4	19
	Domestic	112	12.59	3.25	1	19
	Community	112	8.54	2.93	2	18
	Interpersonal	112	10.13	2.92	1	17
	Play/Leisure	112	10.21	2.63	1	16
	Coping Skills	112	10.10	3.10	1	17
Moderate ID	Receptive	39	7.49	4.54	1	17
	Expressive	39	8.18	4.39	1	16
	Written	39	3.87	3.16	1	11
	Personal	39	8.23	3.62	1	14
	Domestic	39	9.38	3.45	4	16
	Community	39	4.41	3.2	1	13
	Interpersonal	39	7.67	3.26	2	15
	Play/Leisure	39	7.85	3.00	1	13
	Coping Skills	39	7.13	3.74	2	15
Severe ID	Receptive	13	2.38	3.40	1	11
	Expressive	13	2.92	3.97	1	12
	Written	13	1.23	0.60	1	3
	Personal	13	1.54	1.45	1	6
	Domestic	13	3.00	1.87	1	7
	Community	13	1.54	1.127	1	4
	Interpersonal	13	2.92	3.50	1	11
	Play/Leisure	13	2.62	3.15	1	9
	Coping Skills	13	2.92	2.84	1	10

Note. V-score for subdomains (M = 15, SD = 3) according to normative data [1].

**Table A3:** Post hoc comparisons of AB subdomain in different levels of ID.

	Comparison (Level of ID)	Mean Difference	df	t	*p*_(Games-Howwel)_
Communication—Receptive	BIF–Mild ID	1.9	64.2	2.15	0.149
	BIF–Moderate ID	5.36	59.7	6.39	<0.001
	BIF–Severe ID	10.46	17.0	10.13	<0.001
	Mild ID–Moderate ID	4.27	50.2	5.48	<0.001
	Mild ID–Severe ID	9.37	14.3	9.51	<0.001
	Moderate ID–Severe ID	5.10	27.4	4.28	<0.001
Communication—Expressive	BIF–Mild ID	0.816	60.9	1.89	0.242
	BIF–Moderate ID	4.85	56.2	6.13	<0.001
	BIF–Severe ID	10.11	14.7	8.72	<0.001
	Mild ID–Moderate ID	4.3	46.6	5.44	<0.001
	Mild ID–Severe ID	9.29	13.1	8.26	<0.001
	Moderate ID–Severe ID	5.26	22.6	4.2	0.003
Communication—Written	BIF–Mild ID	2.01	54.5	3.90	<0.001
	BIF–Moderate ID	5.86	69.9	8.65	<0.001
	BIF–Severe ID	8.50	39.4	17.76	<0.001
	Mild ID–Moderate ID	3.84	58.5	6.77	<0.001
	Mild ID–Severe ID	6.48	84.7	21.26	<0.001
	Moderate ID–Severe ID	2.64	45.0	4.95	<0.001
Daily Living Skills—Personal	BIF–Mild ID	1.34	64.9	3.34	0.007
	BIF–Moderate ID	5.04	59.1	7.56	<0.001
	BIF–Severe ID	11.73	28.7	22.57	<0.001
	Mild ID–Moderate ID	3.71	50.0	5.95	<0.001
	Mild ID–Severe ID	10.40	20.5	22.55	<0.001
	Moderate ID–Severe ID	6.69	48.1	9.48	<0.001
Daily Living Skills—Domestic	BIF–Mild ID	1.08	78.2	2.21	0.130
	BIF–Moderate ID	4.28	65.1	6.40	<0.001
	BIF–Severe ID	10.67	25.5	16.61	<0.001
	Mild ID–Moderate ID	3.20	63.1	5.7	<0.001
	Mild ID–Severe ID	9.59	21.6	15.90	<0.001
	Moderate ID–Severe ID	6.38	38.8	8.43	<0.001
Daily Living Skills—Community	BIF–Mild ID	2.16	63.6	4.36	<0.001
	BIF–Moderate ID	6.29	69.6	9.89	<0.001
	BIF–Severe ID	9.16	42.2	17.72	<0.001
	Mild ID–Moderate ID	4.13	64.5	7.40	<0.001
	Mild ID–Severe ID	7.00	35.8	16.77	<0.001
	Moderate ID–Severe ID	2.87	49.2	4.98	<0.001
Socialization—Interpersonal	BIF–Mild ID	0.805	78.2	1.84	0.262
	BIF–Moderate ID	3.27	63.5	5.26	<0.001
	BIF–Severe ID	8.02	15.0	7.80	<0.001
	Mild ID–Moderate ID	2.47	60.6	4.18	<0.001
	Mild ID–Severe ID	7.21	14.0	7.15	<0.001
	Moderate ID–Severe ID	4.74	19.4	4.31	0.002
Socialization—Play/Leisure	BIF–Mild ID	0.855	72.9	2.9	0.166
	BIF–Moderate ID	3.21	64.7	5.53	<0.001
	BIF–Severe ID	8.45	15.5	9.6	<0.001
	Mild ID–Moderate ID	2.36	59.5	4.36	<0.001
	Mild ID–Severe ID	7.59	14.0	8.36	<0.001
	Moderate ID–Severe ID	5.23	19.8	5.23	<0.001
Socialization—Coping Skills	BIF–Mild ID	0.690	63.8	1.32	0.556
	BIF–Moderate ID	3.66	66.7	4.95	<0.001
	BIF–Severe ID	7.86	19.7	8.74	<0.001
	Mild ID–Moderate ID	2.97	57.2	4.46	<0.001
	Mild ID–Severe ID	7.18	15.5	8.53	<0.001
	Moderate ID–Severe ID	4.21	27.0	4.25	<0.001

**Table A4:** Paired-samples *t*-test for subdomains in domains of VABS-3 for each level of ID.

Levels of ID		Statistic	df	*p*	Mean Difference	d
BIF	Receptive–Expressive	−0.62	32	0.540	−0.182	−0.11
	Receptive–Written	7.25	32	<0.001	3.121	1.27
	Expressive–Written	7.73	32	<0.001	3.303	1.51
	Personal–Domestic	−1.47	32	0.151	−0.394	−0.26
	Personal–Community	6.83	32	<0.001	2.576	1.19
	Domestic–Community	6.84	32	<0.001	2.970	1.10
	Interpersonal–Play/Leisure	−0.42	32	0.680	−0.121	−0.07
	Interpersonal–Coping Skills	0.47	32	0.639	0.152	0.08
	Play/Leisure–Coping Skills	1.00	32	0.325	0.273	0.17
Mild ID	Receptive–Expressive	−2.63	111	0.010	−0.4554	−0.25
	Receptive–Written	15.1	111	<0.001	4.0446	1.82
	Expressive–Written	20.94	111	<0.001	4.00	1.89
	Personal–Domestic	−3.18	111	0.002	−0.6518	−0.30
	Personal–Community	17.82	111	<0.001	3.18	1.41
	Domestic–Community	16.51	111	<0.001	4.0536	1.98
	Interpersonal–Play/Leisure	−0.35	111	0.725	−0.0714	−0.03
	Interpersonal–Coping Skills	0.17	111	0.870	0.0357	0.02
	Play/Leisure–Coping Skills	0.54	111	0.594	0.1071	0.05
Moderate ID	Receptive–Expressive	−1.96	38	0.057	−0.692	−0.31
	Receptive–Written	6.24	38	<0.001	3.615	0.99
	Expressive–Written	8.11	38	<0.001	4.308	1.83
	Personal–Domestic	−3.52	38	0.001	−1.154	−0.56
	Personal–Community	10.41	38	<0.001	3.821	1.64
	Domestic–Community	17.56	38	<0.001	4.974	2.13
	Interpersonal–Play/Leisure	−0.59	38	0.557	−0.179	−0.1
	Interpersonal–Coping Skills	1.52	38	0.136	0.538	0.24
	Play/Leisure–Coping Skills	2.5	38	0.047	0.718	0.33
Severe ID	Receptive–Expressive	−1.62	12	0.131	−0.538	−0.45
	Receptive–Written	1.47	12	0.166	1.154	0.41
	Expressive–Written	1.78	12	0.100	1.692	0.49
	Personal–Domestic	−3.08	12	0.010	−1.462	−0.85
	Personal–Community	0.00	12	1.000	0.000	0.00
	Domestic–Community	3.96	12	0.002	1.462	1.1
	Interpersonal–Play/Leisure	1.8	12	0.303	0.308	0.3
	Interpersonal–Coping Skills	0.00	12	1.000	0.000	0.00
	Play/Leisure–Coping Skills	−0.69	12	0.502	−0.308	−0.19

**Table A5:** Mean differences of AB domains in each level of ID.

	AB Domain	Mean Difference	*t*-test	df	*p*	Cohen’s d
BIF	Communication–Daily Living Skills	−5.21	−3.157	32	0.003	−0.550
	Communication–Socialization	3.27	1.47	32	0.153	0.255
	Daily Living Skills–Socialization	8.48	5.53	32	<0.001	0.963
Mild ID	Communication–Daily Living Skills	−3.23	−3.60	111	<0.001	−0.340
	Communication–Socialization	1.32	1.09	111	0.278	0.103
	Daily Living Skills–Socialization	4.55	4.75	111	<0.001	0.449
Moderate ID	Communication–Daily Living Skills	−6.436	−3.313	38	0.002	−0.531
	Communication–Socialization	−6.590	−2.87	38	0.007	−0.460
	Daily Living Skills–Socialization	−0.154	−0.115	38	0.909	−0.019
Severe ID	Communication–Daily Living Skills	−1.08	−0.313	12	0.760	−0.087
	Communication–Socialization	−4.38	−3.156	12	0.008	−0.876
	Daily Living Skills–Socialization	−3.31	−0.799	12	0.440	−0.222
